# Supplementary material for: A Whole-Body Physiologically Based Pharmacokinetic Model Characterizing Interplay of OCTs and MATEs in Intestine, Liver and Kidney to Predict Drug-Drug Interactions of Metformin with Perpetrators
Source: Pharmaceutics. 2021 May 11;13(5):698. doi: 10.3390/pharmaceutics13050698 (PMC8151202; doi:10.3390/pharmaceutics13050698)
Supplement: Supplementary file 1 [file pharmaceutics-13-00698-s001.zip › pharmaceutics-1198134-supplement-final.pdf]

pharmaceutics

MDPI

Supplementary Materials: A Whole-Body Physiologically Based Pharmacokinetic Model Characterizing Interplay of OCTs and MATEs in Intestine, Liver and Kidney to Predict Drug-Drug Interactions of Metformin with Perpetrators

Yiting Yang, Zexin Zhang, Ping Li, Weimin Kong, Xiaodong Liu and Li Liu

1. Physiological parameters for human used in the PBPK model.

Table S1. Physiological parameters for human used in the PBPK model.

Tissue	V (mL) [1,2]	Q (mL/min) [1]	Transit rate constant (min^{-1}) [3,4]	pH [5]
Lung	1170	5600	/	
Kidneys	280	1240	/	
Heart	310	240	/	
Liver	1690	300	/	
Muscle	35,000	750	/	
Skin	7800	300	/	
Brain	1450	700	/	
Adipose	10,000	260	/	
Rob	5100	592	/	
Spleen	190	80	/	
Artery	1730	5600	/	
Venous	3470	5600	/	
Stomach	160	38	0.0462	
Duodenum	70	118	0.0462	5.67
Jejunum	209	413	0.012	6.51
Ileum	139	244	0.0058	7.42
Cecum	116	44	0.004	NA
Colon	1116	281	0.0013	6.47

2. Ratios of concentration in tissue to plasma for building PBPK model.

Table S2. Ratios of concentration in tissue to plasma for metformin (Met), cimetidine (Cim), pyrimethamine (Pyr), trimethoprim (Tri), ondansetron (Ond), rabeprazole (Rab) and verapamil (Ver) [6].

Tissue	Met	Cim	Pyr	Tri	Ond	Rab	Ver
pKa	11.8	6.9	7.77	7.16	7.34	5	8.5
LogP	−1.43	0.48	2.62	1.28	2.56	2.3	3.79
Adipose	0.18	1.89	6.8	3.8	39.02	0.08	12.95
Liver	1.6	1.46	7.5	1.9	1.17	0.10	60.55
Muscle	1.7	1.18	1.23	0.98	0.74	0.08	7.58
Lung	1.46	2.35	2.4	2.04	1.48	0.22	14.24
Kidney	1.49	1.58	6.8	1.9	1.12	0.14	54.70
Brain	1.72	1.62	1.9	1.53	1.99	0.06	8.69
Heart	1.47	1.42	2.7	1.34	1.06	0.17	19.27
Intestine	1.5	1.18	3.1	1.3	2.17	0.17	21.87
Skin	1.11	2.31	2.1	1.94	3.12	0.29	12.49

Spleen	1.66	2.02	3.6	1.89	0.73	0.11	25.06
Stomach	1.5	1.18	3.1	1.3	0.74	0.08	21.87
Rest	1.2	0.01	0.01	1	0.01	0.01	0.01

3. The investigation of saturable metformin absorption

Table S3 and S4 are the observed and predicted pharmacokinetic parameters of metformin for different dosage, respectively. Simulation demonstrated that non-linear pharmacokinetics of metformin was attributed to transporter-mediated intestinal absorption.

Table S3. The observed pharmacokinetic parameters of metformin for different dosage.

Dose (mg)	Ref.	C_{max} (ug/mL)	AUC (h·ug/mL)	Dose/AUC
250	[7]	0.59	4.26	58.7
250	[8]	0.78	4.98	50.2
250	[9]	0.74	4.89	51.1
500	[10]	1.1	5.8	86.2
500	[11]	1.55	9.08	55.1
500	[12]	1.19	6.58	76.0
500	[13]	1.21	7.40	67.6
500	[14]	0.77	5.96	83.9
500	[15]	1.3	6.8	73.5
500	[16]	2.1	16.9	29.6
500	[17]	1.14	6.5	76.9
500	[18]	1.05	6.3	79.4
500	[19]	2.0	12.1	41.32
500	[20]	0.84	6.3	79.4
500	[21]	0.99	6.55	76.3
750	[22]	1.46	6.52	115.0
750	[23]	1.51	8.22	103.9
750	[24]	1.5	9.4	79.8
750	[25]	4.2	24.69	30.4
750	[26]	1.38	9.17	81.8
850	[27]	1.3	7.7	110.4
850	[28]	1.2	7.2	118.1
850	[29]	1.81	11.4	74.6
850	[30]	1.17	6.69	127.1
850	[31]	2.28	15.2	55.9
850	[32]	1.26	14.2	59.9
1000	[33]	2.0	13.2	75.8
1000	[34]	1.8	11.1	90.1
1000	[35]	2.06	12.41	80.58
1000	[36]	1.35	10.3	97.1
1000	[37]	2.35	12.81	78.1
1000	[38]	2.19	14	71.4
1000	[39]	1.79	12.57	79.5
1000	[40]	1.18	7.95	125.8
1000	[41]	1.46	10.6	94.3

Table S4. The predicted pharmacokinetic parameters of metformin for different dosage.

Dose (mg)	C_{max} (ug/mL)	AUC (h·ug/mL)	Dose/AUC
250	0.67	4.80	52.06
500	0.95	7.68	65.11
750	1.22	10.19	73.61
850	1.32	11.15	76.26
1000	1.47	12.55	79.68

4. Quantitatively predicted disposition kinetics for perpetrators

Table S5. The observed pharmacokinetic parameters of perpetrators in different references.

Drug	Ref.	Dose (mg)	C_{max} (μg/mL)		AUC_{0-t} (μg·h/mL)	
			Pre	Obs	Pre	Obs
Cimetidine	[42]	400	2.06	2.20	9.17	8.03
	[43]	300	1.53	1.53	6.68	5.22
	[44]	400	2.06	1.5	8.90	10.4
	[45]	400	2.06	1.93	8.61	8.4
	[45]	800	4.11	3.7	17.28	17.89
	[46]	800	4.11	2.47	16.76	11.49
Pyrimethamine	[47]	50	0.5	0.76	47.66	76
	[48]	75	0.86	0.86	51.78	124.6
	[49]	75	0.86	0.38	106.42	79.42
Trimethoprim	[50]	210	2.18	2.35	28.26	37.1
	[51]	1400	13.76	12.78	302.34	299.31
Ondansetron	[52]	8	0.046	0.0272	0.203	0.198
	[53]	8	0.046	0.037	0.233	0.254
	[54]	8	0.046	0.033	0.253*	0.287*
Rabeprazole	[55]	40	0.40	0.502	1.26	1.315
	[55]	40	0.40	0.444	1.26	1.332
	[56]	20	0.20	0.252	0.62	0.575
	[57]	10	0.10	0.15	0.29	0.36
	[58]	20	0.20	0.47	0.62	0.90
	[56]	20	0.20	0.25	0.62	0.58
Verapamil	[59]	40	0.050	0.033	0.28	0.22
	[60]	80	0.099	0.13	0.564	0.387
	[61]	80	0.099	0.082	0.439	0.17
	[62]	160	0.20	0.16	0.82	0.80

* AUC is calculated from 0 to ∞.

5. The administration dosage for coadministration of metformin and perpetrators

The administration dosage of metformin and 6 perpetrators, cimetidine, pyrimethamine, trimethoprim, ondansetron, rabeprazole and verapamil were shown in Table S6.

Table S6. The administration schedule for metformin and perpetrators.

Perpetrator	Ref.	administration schedule
Cimetidine	[7]	The subjects received a single daily oral dose of 250 mg metformin in addition 400 mg cimetidine twice daily (2 × 200 mg)
Cimetidine	[12]	The subjects received a single oral dose of 400 mg cimetidine and then a single oral dose of 500 mg metformin 2 h later

py-rimetham-ine	[63]	The subjects received 250 mg metformin 1 h after receiving 50 mg pyrimethamine
py-rimetham-ine	[63]	The subjects received 0.1 mg metformin 1 h after receiving 50 mg pyrimethamine
py-rimetham-ine	[14]	500 mg metformin was administered with 50 mg pyrimethamine
trime-thoprim	[15]	Metformin 500 mg and trimethoprim 200 mg were administered orally at midnight and at 20:00 on the day before pharmacokinetic evaluation study. On study day, trimethoprim 200 mg was given half an hour before administration of 500 mg metformin
trime-thoprim	[30]	850 mg metformin and 200 mg trimethoprim were administered orally in the evening (20:00) on the day before pharmacokinetic evaluation study and in the morning of study day (8:00)
on-dansetron	[31]	The subjects received 850 mg metformin 1 h after receiving 8 mg ondansetron
rabeprazole	[10]	The first dose of metformin (1000 mg) was taken orally on the day before study day at 20:00 PM, and the second dose (750 mg) metformin together with rabeprazole or placebo was given on study day at 8:00 AM with a 12 h interval
rabeprazole	[25]	At 8 PM the participants were given metformin (750 mg by mouth). The following day at 8 AM the subjects were given metformin 500 mg (by mouth) with rabeprazole
verapamil	[23]	750 mg metformin was administered with 180 mg verapamil

Reference

1. Davies, B.; Morris, T. Physiological parameters in laboratory animals and humans. *Pharm Res* **1993**, *10*, 1093–1095, doi:10.1023/a:1018943613122.
2. DeSesso, J.M.; Jacobson, C.F. Anatomical and physiological parameters affecting gastrointestinal absorption in humans and rats. *Food Chem Toxicol* **2001**, *39*, 209–228, doi:10.1016/s0278-6915(00)00136-8.
3. Perdaems, N.; Blasco, H.; Vinson, C.; Chenel, M.; Whalley, S.; Cazade, F.; Bouzom, F. Predictions of metabolic drug-drug interactions using physiologically based modelling: Two cytochrome P450 3A4 substrates coadministered with ketoconazole or verapamil. *Clin Pharmacokinet* **2010**, *49*, 239–258, doi:10.2165/11318130-000000000-00000.
4. Heikkinen, A.T.; Baneyx, G.; Caruso, A.; Parrott, N. Application of PBPK modeling to predict human intestinal metabolism of CYP3A substrates - an evaluation and case study using GastroPlus. *Eur J Pharm Sci* **2012**, *47*, 375–386, doi:10.1016/j.ejps.2012.06.013.
5. Abuhelwa, A.Y.; Foster, D.J.R.; Upton, R.N. A Quantitative Review and Meta-Models of the Variability and Factors Affecting Oral Drug Absorption-Part I: Gastrointestinal pH. *AAPS J* **2016**, *18*, 1309–1321, doi:10.1208/s12248-016-9952-8.
6. Rodgers, T.; Rowland, M. Physiologically based pharmacokinetic modelling 2: predicting the tissue distribution of acids, very weak bases, neutrals and zwitterions. *J Pharm Sci* **2006**, *95*, 1238–1257, doi:10.1002/jps.20502.
7. Somogyi, A.; Stockley, C.; Keal, J.; Rolan, P.; Bochner, F. Reduction of metformin renal tubular secretion by cimetidine in man. *Br J Clin Pharmacol* **1987**, *23*, 545-551, doi:10.1111/j.1365–2125.1987.tb03090.x.
8. Ｒong，, G.; Zhong，, M.Y.; Xia，, Z.H.; Yuan，, W.Z.; Hong，, Z.W.; Long, Y.H. Bioequivalence of metformin hydrochloride tablets in healthy Chinese subjects. *Chin J Clin Pharmacol* **2021**, *37*, 222–226.
9. CHEN Li; ZHANG Xin; LU Xing; XIAO Chun yan; QIAO Ke; CHEN Xiao wu; tong, L.J. Bioequivalence of metformin hydrochloride in Chinese healthy subjects. *Chin J Clin Pharmacol* **2019**, *35*, 854-857, doi:10. 13699 /j. cnki. 1001 － 6821. 2019. 09. 009.

10. Kim, A.; Chung, I.; Yoon, S.H.; Yu, K.S.; Lim, K.S.; Cho, J.Y.; Lee, H.; Jang, I.J.; Chung, J.Y. Effects of proton pump inhibitors on metformin pharmacokinetics and pharmacodynamics. *Drug Metab Dispos* **2014**, *42*, 1174–1179, doi:10.1124/dmd.113.055616.
11. Pentikainen, P.J.; Neuvonen, P.J.; Penttila, A. Pharmacokinetics of metformin after intravenous and oral administration to man. *Eur J Clin Pharmacol* **1979**, *16*, 195–202, doi:10.1007/BF00562061.
12. Wang, Z.J.; Yin, O.Q.; Tomlinson, B.; Chow, M.S. OCT2 polymorphisms and in-vivo renal functional consequence: studies with metformin and cimetidine. *Pharmacogenet Genomics* **2008**, *18*, 637–645, doi:10.1097/FPC.0b013e328302cd41.
13. Garza-Ocanas, L.; Gonzalez-Canudas, J.; Tamez-de la, O.E.; Badillo-Castaneda, C.; Gomez-Meza, M.V.; Romero-Antonio, Y.; Molina-Perez, A.; Amador-Hernandez, A.G. Comparative Bioavailability of Metformin Hydrochloride Oral Solution Versus Metformin Hydrochloride Tablets in Fasting Mexican Healthy Volunteers. *Adv Ther* **2019**, *36*, 407–415, doi:10.1007/s12325-018-0853-3.
14. Oh, J.; Chung, H.; Park, S.I.; Yi, S.J.; Jang, K.; Kim, A.H.; Yoon, J.; Cho, J.Y.; Yoon, S.H.; Jang, I.J., et al. Inhibition of the multidrug and toxin extrusion (MATE) transporter by pyrimethamine increases the plasma concentration of metformin but does not increase antihyperglycaemic activity in humans. *Diabetes Obes Metab* **2016**, *18*, 104–108, doi:10.1111/dom.12577.
15. Grun, B.; Kiessling, M.K.; Burhenne, J.; Riedel, K.D.; Weiss, J.; Rauch, G.; Haefeli, W.E.; Czock, D. Trimethoprim-metformin interaction and its genetic modulation by OCT2 and MATE1 transporters. *Br J Clin Pharmacol* **2013**, *76*, 787–796, doi:10.1111/bcp.12079.
16. McCreight, L.J.; Stage, T.B.; Connelly, P.; Lonergan, M.; Nielsen, F.; Prehn, C.; Adamski, J.; Brosen, K.; Pearson, E.R. Pharmacokinetics of metformin in patients with gastrointestinal intolerance. *Diabetes Obes Metab* **2018**, *20*, 1593-1601, doi:10.1111/dom.13264.
17. Jang, K.; Chung, H.; Yoon, J.S.; Moon, S.J.; Yoon, S.H.; Yu, K.S.; Kim, K.; Chung, J.Y. Pharmacokinetics, Safety, and Tolerability of Metformin in Healthy Elderly Subjects. *J Clin Pharmacol* **2016**, *56*, 1104–1110, doi:10.1002/jcph.699.
18. Groen-Wijnberg, M.; van Dijk, J.; Krauwinkel, W.; Kerbusch, V.; Meijer, J.; Tretter, R.; Zhang, W.; van Gelderen, M. Pharmacokinetic Interactions Between Mirabegron and Metformin, Warfarin, Digoxin or Combined Oral Contraceptives. *Eur J Drug Metab Pharmacokinet* **2017**, *42*, 417–429, doi:10.1007/s13318-016-0350-5.
19. Te Brake, L.H.M.; Yunivita, V.; Livia, R.; Soetedjo, N.; van Ewijk-Beneken Kolmer, E.; Koenderink, J.B.; Burger, D.M.; Santoso, P.; van Crevel, R.; Alisjahbana, B., et al. Rifampicin Alters Metformin Plasma Exposure but Not Blood Glucose Levels in Diabetic Tuberculosis Patients. *Clin Pharmacol Ther* **2019**, *105*, 730-737, doi:10.1002/cpt.1232.
20. Zaharenko, L.; Kalnina, I.; Geldnere, K.; Konrade, I.; Grinberga, S.; Zidzik, J.; Javorsky, M.; Lejnieks, A.; Nikitina-Zake, L.; Fridmanis, D., et al. Single nucleotide polymorphisms in the intergenic region between metformin transporter OCT2 and OCT3 coding genes are associated with short-term response to metformin monotherapy in type 2 diabetes mellitus patients. *Eur J Endocrinol* **2016**, *175*, 531-540, doi:10.1530/Eje-16-0347.
21. Kim, S.; Jang, I.J.; Shin, D.; Shin, D.S.; Yoon, S.; Lim, K.S.; Yu, K.S.; Li, J.; Zhang, H.; Liu, Y., et al. Investigation of bioequivalence of a new fixed-dose combination of acarbose and metformin with the corresponding loose combination as well as the drug-drug interaction potential between both drugs in healthy adult male subjects. *J Clin Pharm Ther* **2014**, *39*, 424-431, doi:10.1111/jcpt.12166.
22. Wen, J.; Zeng, M.; Liu, Z.; Zhou, H.; Xu, H.; Huang, M.; Zhang, W. The influence of telmisartan on metformin pharmacokinetics and pharmacodynamics. *J Pharmacol Sci* **2019**, *139*, 37-41, doi:10.1016/j.jphs.2018.11.007.
23. Cho, S.K.; Kim, C.O.; Park, E.S.; Chung, J.Y. Verapamil decreases the glucose-lowering effect of metformin in healthy volunteers. *Br J Clin Pharmacol* **2014**, *78*, 1426-1432, doi:10.1111/bcp.12476.

24. Cho, S.K.; Yoon, J.S.; Lee, M.G.; Lee, D.H.; Lim, L.A.; Park, K.; Park, M.S.; Chung, J.Y. Rifampin enhances the glucose-lowering effect of metformin and increases OCT1 mRNA levels in healthy participants. *Clin Pharmacol Ther* **2011**, *89*, 416-421, doi:10.1038/clpt.2010.266.
25. Liu, G.; Wen, J.; Guo, D.; Wang, Z.; Hu, X.; Tang, J.; Liu, Z.; Zhou, H.; Zhang, W. The effects of rabeprazole on metformin pharmacokinetics and pharmacodynamics in Chinese healthy volunteers. *J Pharmacol Sci* **2016**, *132*, 244-248, doi:10.1016/j.jphs.2016.04.016.
26. Shibata, M.; Toyoshima, J.; Kaneko, Y.; Oda, K.; Nishimura, T. A drug-drug interaction study to evaluate the impact of peficitinib on OCT1-and MATE1-mediated transport of metformin in healthy volunteers. *European Journal of Clinical Pharmacology* **2020**, *76*, 1135-1141, doi:10.1007/s00228-020-02876-2.
27. Shu, Y.; Brown, C.; Castro, R.A.; Shi, R.J.; Lin, E.T.; Owen, R.P.; Sheardown, S.A.; Yue, L.; Burchard, E.G.; Brett, C.M., et al. Effect of genetic variation in the organic cation transporter 1, OCT1, on metformin pharmacokinetics. *Clin Pharmacol Ther* **2008**, *83*, 273-280, doi:10.1038/sj.clpt.6100275.
28. Rocha, J.F.; Vaz-da-Silva, M.; Almeida, L.; Falcao, A.; Nunes, T.; Santos, A.T.; Martins, F.; Fontes-Ribeiro, C.; Macedo, T.; Soares-da-Silva, P. Effect of eslicarbazepine acetate on the pharmacokinetics of metformin in healthy subjects. *Int J Clin Pharmacol Ther* **2009**, *47*, 255-261, doi:10.5414/cpp47255.
29. Morrissey, K.M.; Stocker, S.L.; Chen, E.C.; Castro, R.A.; Brett, C.M.; Giacomini, K.M. The Effect of Nizatidine, a MATE2K Selective Inhibitor, on the Pharmacokinetics and Pharmacodynamics of Metformin in Healthy Volunteers. *Clin Pharmacokinet* **2016**, *55*, 495-506, doi:10.1007/s40262-015-0332-9.
30. Muller, F.; Pontones, C.A.; Renner, B.; Mieth, M.; Hoier, E.; Auge, D.; Maas, R.; Zolk, O.; Fromm, M.F. N(1)-methylnicotinamide as an endogenous probe for drug interactions by renal cation transporters: studies on the metformin-trimethoprim interaction. *Eur J Clin Pharmacol* **2015**, *71*, 85-94, doi:10.1007/s00228-014-1770-2.
31. Li, Q.; Yang, H.; Guo, D.; Zhang, T.; Polli, J.E.; Zhou, H.; Shu, Y. Effect of Ondansetron on Metformin Pharmacokinetics and Response in Healthy Subjects. *Drug Metab Dispos* **2016**, *44*, 489-494, doi:10.1124/dmd.115.067223.
32. Hibma, J.E.; Zur, A.A.; Castro, R.A.; Wittwer, M.B.; Keizer, R.J.; Yee, S.W.; Goswami, S.; Stocker, S.L.; Zhang, X.; Huang, Y., et al. The Effect of Famotidine, a MATE1-Selective Inhibitor, on the Pharmacokinetics and Pharmacodynamics of Metformin. *Clin Pharmacokinet* **2016**, *55*, 711-721, doi:10.1007/s40262-015-0346-3.
33. Dawra, V.K.; Cutler, D.L.; Zhou, S.; Krishna, R.; Shi, H.; Liang, Y.; Alvey, C.; Hickman, A.; Saur, D.; Terra, S.G., et al. Assessment of the Drug Interaction Potential of Ertugliflozin With Sitagliptin, Metformin, Glimepiride, or Simvastatin in Healthy Subjects. *Clin Pharmacol Drug Dev* **2019**, *8*, 314-325, doi:10.1002/cpdd.472.
34. Padwal, R.S.; Gabr, R.Q.; Sharma, A.M.; Langkaas, L.A.; Birch, D.W.; Karmali, S.; Brocks, D.R. Effect of gastric bypass surgery on the absorption and bioavailability of metformin. *Diabetes Care* **2011**, *34*, 1295-1300, doi:10.2337/dc10-2140.
35. Smolders, E.J.; Colbers, A.; de Kanter, C.; Velthoven-Graafland, K.; Wolberink, L.T.; van Ewijk-Beneken Kolmer, N.; Drenth, J.P.H.; Aarnoutse, R.E.; Tack, C.J.; Burger, D.M. Metformin and daclatasvir: absence of a pharmacokinetic-pharmacodynamic drug interaction in healthy volunteers. *Br J Clin Pharmacol* **2017**, *83*, 2225-2234, doi:10.1111/bcp.13323.
36. Sanchez, R.I.; Yee, K.L.; Fan, L.; Cislak, D.; Martell, M.; Jordan, H.R.; Iwamoto, M.; Khalilieh, S. Evaluation of the Pharmacokinetics of Metformin Following Coadministration With Doravirine in Healthy Volunteers. *Clin Pharmacol Drug Dev* **2020**, *9*, 107-114, doi:10.1002/cpdd.685.
37. Conde-Carmona, I.; Garcia-Medina, S.; Jimenez-Vargas, J.M.; Martinez-Munoz, A.; Lee, S.H. Pharmacokinetic Interactions Between Gemigliptin and Metformin, and Potential Differences in the Pharmacokinetic Profile of Gemigliptin Between the Mexican and Korean Populations: A Randomized, Open-label Study in Healthy Mexican Volunteers. *Clinical Therapeutics* **2018**, *40*, 1729-1740, doi:10.1016/j.clinthera.2018.08.015.

38. Christensen, M.M.H.; Hojlund, K.; Hother-Nielsen, O.; Stage, T.B.; Damkier, P.; Beck-Nielsen, H.; Brosen, K. Steady-state pharmacokinetics of metformin is independent of the OCT1 genotype in healthy volunteers. *European Journal of Clinical Pharmacology* **2015**, *71*, 691-697, doi:10.1007/s00228-015-1853-8.
39. Yi, L.; Zhang, H.; Zhang, J.W.; You, X.M.; Ning, Z.Q.; Yu, J.; Qian, L.F.; Miao, L.Y. Study on Drug-Drug Interactions Between Chiglitazar, a Novel PPAR Pan-Agonist, and Metformin Hydrochloride in Healthy Subjects. *Clin Pharm Drug Dev* **2019**, *8*, 934-941, doi:10.1002/cpdd.668.
40. Lesko, L.J.; Offman, E.; Brew, C.T.; Garza, D.; Benton, W.; Mayo, M.R.; Romero, A.; Du Mond, C.; Weir, M.R. Evaluation of the Potential for Drug Interactions With Patiromer in Healthy Volunteers. *J Cardiovasc Pharm T* **2017**, *22*, 434-446, doi:10.1177/1074248417691135.
41. Kuhlmann, I.; Pedersen, S.A.; Esbech, P.S.; Stage, T.B.; Christensen, M.M.H.; Brosen, K. Using a limited sampling strategy to investigate the interindividual pharmacokinetic variability in metformin: A large prospective trial. *Brit J Clin Pharmaco* **2020**, 10.1111/bcp.14591, doi:10.1111/bcp.14591.
42. Jantratid, E.; Prakongpan, S.; Amidon, G.L.; Dressman, J.B. Feasibility of biowaiver extension to biopharmaceutics classification system class III drug products: cimetidine. *Clin Pharmacokinet* **2006**, *45*, 385-399, doi:10.2165/00003088-200645040-00004.
43. Walkenstein, S.S.; Dubb, J.W.; Randolph, W.C.; Westlake, W.J.; Stote, R.M.; Intoccia, A.P. Bioavailability of cimetidine in man. *Gastroenterology* **1978**, *74*, 360-365.
44. Grahnen, A.; von Bahr, C.; Lindstrom, B.; Rosen, A. Bioavailability and pharmacokinetics of cimetidine. *Eur J Clin Pharmacol* **1979**, *16*, 335-340, doi:10.1007/BF00605632.
45. Bodemar, G.; Norlander, B.; Walan, A. Pharmacokinetics of cimetidine after single doses and during continuous treatment. *Clin Pharmacokinet* **1981**, *6*, 306-315, doi:10.2165/00003088-198106040-00005.
46. Klein-Schwartz, W.; Doyon, S.; Dowling, T. Drug adsorption efficacy and palatability of a novel charcoal cookie formulation. *Pharmacotherapy* **2010**, *30*, 888-894, doi:10.1592/phco.30.9.888.
47. Mansor, S.M.; Navaratnam, V.; Mohamad, M.; Hussein, S.; Kumar, A.; Jamaludin, A.; Wernsdorfer, W.H. Single dose kinetic study of the triple combination mefloquine/sulphadoxine/pyrimethamine (Fansimef) in healthy male volunteers. *Br J Clin Pharmacol* **1989**, *27*, 381-386, doi:10.1111/j.1365-2125.1989.tb05381.x.
48. Minzi, O.M.; Gupta, A.; Haule, A.F.; Kagashe, G.A.; Massele, A.Y.; Gustafsson, L.L. Lack of impact of artesunate on the disposition kinetics of sulfadoxine/pyrimethamine when the two drugs are concomitantly administered. *Eur J Clin Pharmacol* **2007**, *63*, 457-462, doi:10.1007/s00228-007-0278-4.
49. Nyunt, M.M.; Adam, I.; Kayentao, K.; van Dijk, J.; Thuma, P.; Mauff, K.; Little, F.; Cassam, Y.; Guirou, E.; Traore, B., et al. Pharmacokinetics of sulfadoxine and pyrimethamine in intermittent preventive treatment of malaria in pregnancy. *Clin Pharmacol Ther* **2010**, *87*, 226-234, doi:10.1038/clpt.2009.177.
50. Hoppu, K.; Tuomisto, J.; Koskimies, O.; Simell, O. Food and guar decrease absorption of trimethoprim. *Eur J Clin Pharmacol* **1987**, *32*, 427-429, doi:10.1007/BF00543981.
51. Hruska, M.W.; Frye, R.F. Determination of trimethoprim in low-volume human plasma by liquid chromatography. *J Chromatogr B Analyt Technol Biomed Life Sci* **2004**, *807*, 301-305, doi:10.1016/j.jchromb.2004.04.021.
52. Villikka, K.; Kivisto, K.T.; Neuvonen, P.J. The effect of rifampin on the pharmacokinetics of oral and intravenous ondansetron. *Clin Pharmacol Ther* **1999**, *65*, 377-381, doi:10.1016/S0009-9236(99)70130-X.
53. Zhu, Y.; Zhang, Q.; Zou, J.; Wan, M.; Zhao, Z.; Zhu, J. Pharmacokinetics and bioavailability study of two ondansetron oral soluble film formulations in fasting healthy male Chinese volunteers. *Drug Des Devel Ther* **2015**, *9*, 4621-4629, doi:10.2147/DDDT.S86415.

54. Dychter, S.S.; Harrigan, R.; Bahn, J.D.; Printz, M.A.; Sugarman, B.J.; DeNoia, E.; Haughey, D.B.; Fellows, D.; Maneval, D.C. Tolerability and pharmacokinetic properties of ondansetron administered subcutaneously with recombinant human hyaluronidase in minipigs and healthy volunteers. *Clin Ther* **2014**, *36*, 211-224, doi:10.1016/j.clinthera.2013.12.013.
55. Chen, J.; Jiang, W.M.; Gao, X.L.; Jiang, X.; Zhang, Q.Z.; Zheng, Z.H. Bioequivalence evaluation of two rabeprazole enteric coated formulations in healthy Chinese volunteers. *Eur J Drug Metab Pharmacokinet* **2004**, *29*, 103-106, doi:10.1007/BF03190583.
56. Noubarani, M.; Keyhanfar, F.; Motevalian, M.; Mahmoudian, M. Improved HPLC method for determination of four PPIs, omeprazole, pantoprazole, lansoprazole and rabeprazole in human plasma. *J Pharm Pharm Sci* **2010**, *13*, 1-10, doi:10.18433/j3gp4q.
57. Thyssen, A.; Solanki, B.; Treem, W. Randomized, open-label, single-dose, crossover, relative bioavailability study in healthy adults, comparing the pharmacokinetics of rabeprazole granules administered using soft food or infant formula as dosing vehicle versus suspension. *Clin Ther* **2012**, *34*, 1636-1645, doi:10.1016/j.clinthera.2012.06.008.
58. Arai, K.; Takeuchi, Y.; Watanabe, H.; Tsukurimichi, A.; Uchida, N.; Imawari, M. Prokinetics influence the pharmacokinetics of rabeprazole. *Digestion* **2008**, *78*, 67-71, doi:10.1159/000165351.
59. Sawicki, W. Pharmacokinetics of verapamil and norverapamil from controlled release floating pellets in humans. *Eur J Pharm Biopharm* **2002**, *53*, 29-35, doi:10.1016/s0939-6411(01)00189-8.
60. Dadashzadeh, S.; Javadian, B.; Sadeghian, S. The effect of gender on the pharmacokinetics of verapamil and norverapamil in human. *Biopharm Drug Dispos* **2006**, *27*, 329-334, doi:10.1002/bdd.512.
61. Hamann, S.R.; Blouin, R.A.; McAllister, R.G., Jr. Clinical pharmacokinetics of verapamil. *Clin Pharmacokinet* **1984**, *9*, 26-41, doi:10.2165/00003088-198409010-00002.
62. Meredith, P.A.; Elliott, H.L.; Pasanisi, F.; Kelman, A.W.; Sumner, D.J.; Reid, J.L. Verapamil pharmacokinetics and apparent hepatic and renal blood flow. *Br J Clin Pharmacol* **1985**, *20*, 101-106, doi:10.1111/j.1365-2125.1985.tb05038.x.
63. Kusuhara, H.; Ito, S.; Kumagai, Y.; Jiang, M.; Shiroshita, T.; Moriyama, Y.; Inoue, K.; Yuasa, H.; Sugiyama, Y. Effects of a MATE protein inhibitor, pyrimethamine, on the renal elimination of metformin at oral microdose and at therapeutic dose in healthy subjects. *Clin Pharmacol Ther* **2011**, *89*, 837-844, doi:10.1038/clpt.2011.36.
